# Study of the Mechanisms of Radiation Softening and Swelling upon Irradiation of TiTaNbV Alloys with He^2+^ Ions with an Energy of 40 keV

**DOI:** 10.3390/ma16114031

**Published:** 2023-05-28

**Authors:** Sholpan G. Giniyatova, Kayrat K. Kadyrzhanov, Dmitriy I. Shlimas, Daryn B. Borgekov, Vladimir V. Uglov, Artem L. Kozlovskiy, Maxim V. Zdorovets

**Affiliations:** 1 Engineering Profile Laboratory, L.N. Gumilyov Eurasian National University, Astana 010008, Kazakhstan; giniyatova_shg@enu.kz (S.G.G.); shlimas@mail.ru (D.I.S.); borgekov@mail.ru (D.B.B.); mzdorovets@gmail.com (M.V.Z.); 2 Laboratory of Solid State Physics, The Institute of Nuclear Physics, Almaty 050032, Kazakhstan; 3 Department of General Physics, Satbayev University, Almaty 050032, Kazakhstan; uglov@bsu.by; 4 Department of Solid State Physics, Belarusian State University, 220050 Minsk, Belarus; 5 Department of Intelligent Information Technologies, Ural Federal University, Yekaterinburg 620075, Russia

**Keywords:** swelling, radiation embrittlement, helium swelling, high entropy alloys, hardness

## Abstract

This paper presents simulation results of the ionization losses of incident He^2+^ ions with an energy of 40 keV during the passage of incident ions in the near-surface layer of alloys based on TiTaNbV with a variation of alloy components. For comparison, data on the ionization losses of incident He^2+^ ions in pure niobium, followed by the addition of vanadium, tantalum, and titanium to the alloy in equal stoichiometric proportions, are presented. With the use of indentation methods, the dependences of the change in the strength properties of the near-surface layer of alloys were determined. It was established that the addition of Ti to the composition of the alloy leads to an increase in resistance to crack resistance under high-dose irradiation, as well as a decrease in the degree of swelling of the near-surface layer. During tests on the thermal stability of irradiated samples, it was found that swelling and degradation of the near-surface layer of pure niobium affects the rate of oxidation and subsequent degradation, while for high-entropy alloys, an increase in the number of alloy components leads to an increase in resistance to destruction.

## 1. Introduction

As is known, the safe development of the nuclear industry, as well as the exploration of outer space using spacecraft, requires new types of structural materials with increased radiation resistance. At the same time, the use of traditional types of steels and alloys in modern realities of the operation of structural materials in high-temperature nuclear reactors is limited by the temperature conditions of the core, as well as accelerated processes of accumulation of radiation damage during prolonged exposure [1,2,3]. In the case of long-term high-temperature irradiation, traditional methods of stability increase by alloying steels or creating nanostructural inclusions in them, which make it possible to change the properties of binary alloys, are not always effective [4,5]. Under high-dose irradiation, nanostructured inclusions, which are mostly metastable, can destabilize the irradiated material due to thermal effects, leading to accelerated destruction and embrittlement. In this case, a decrease in the strength characteristics of materials can have a negative impact on maintaining the stability of the performance of materials, as well as resistance to long-term operation. At the same time, long-term radiation exposure leading to embrittlement of the near-surface layer leads to a destructive change in the properties of materials and in some cases, uncontrolled swelling, leading to elongation, which can lead to catastrophic consequences in the reactor zone [6,7]. Additionally, an important factor affecting the processes of accumulation of radiation damages and their consequences are sharp temperature drops that can occur when changing the operating mode of the core, quick removal of rods, etc. In this case, the material is subjected to rapid heating or sharp cooling, which can lead to additional destabilization of accumulated radiation damage due to a change in the temperature gradient that can affect the mobility of radiation-induced damage [8,9,10]. In the case of gas-filled bubbles, a change in the temperature gradient can lead to destabilization, as well as the acceleration of agglomeration processes, which in turn will lead to an increase in their volume, and as a result, an increase in mechanical stresses caused by stretching of the region near the formed gas-filled bubbles [11,12,13]. A change in the stress concentration and deformation distortions in the near-surface layer can lead to accelerated destabilization of the material and, as a result, its softening [14,15].

Interest in high-entropy alloys in the past few years has been due to the great prospects for their use as structural materials requiring operation at elevated temperatures or mechanical stress, as well as radiation exposure. At the same time, in several works [16,17,18], it was shown that the variation of the components of high-entropy alloys leads to a change in the resistance to radiation-induced swelling and subsequent embrittlement and in some cases, an increase in structural stability in comparison with classical nuclear materials subjected to similar radiation exposure. The increased stability is primarily due to the fact that when varying the alloy components, additional grain boundaries are created, as well as, in some cases, the formation of a high density of dislocations and vacancy defects, the presence of which causes the effects of inhibition of the ability to agglomerate implanted ions in the near-surface layer [19,20]. However, despite the rather large number of different studies related to the study of radiation resistance and resistance to radiation-induced embrittlement and softening for high-entropy alloys, there are still many unresolved questions concerning both the fundamental aspects of the study of radiation resistance processes and practical tests aimed at the study of the relationship between the radiation damage degree and changes in structural and strength properties.

The aim of this study is to evaluate the variation in the composition of the high-entropy TiTaNbV alloy for resistance to radiation damage during helium irradiation, as well as to subsequent degradation-deformation changes in the near-surface layer. Interest in studies of mechanical properties and their changes as a result of external influences, in particular, radiation damage, is due to the need to understand the processes of destruction of the near-surface layer. The results of the studies obtained will make it possible to assess how resistant these alloys are to the accumulation of radiation damage, which will make it possible to determine the maximum allowable mechanical loads on the material during their operation. Additionally, the study of the mechanical properties of high-entropy alloys has received quite a lot of attention recently in view of the expansion of the range of their practical application and the possibility of operating under conditions of increased mechanical pressure, aggressive media, or background radiation. For example, in [21], the authors considered the effects of hardening associated with a variation in the composition of a high-entropy alloy that has great prospects for use as structural material. Additionally, in [22], the factors affecting structural distortions and hardening of high-entropy alloys, which are highly promising in their use as materials with high strength and thermal stability, are studied in detail. As shown in several works [23,24,25], the presence of several components in alloys, and their variation, results in an increase in hardening due to the occurrence of boundary effects associated with different grains or a dendritic structure [22]. So, for example, in works [24,25], it is shown that the variation of alloy components leads to an increase in resistance to external influences due to boundary effects, as well as structural distortions.

## 2. Materials and Methods

### 2.1. Initial Samples

High-entropy alloys based on TiTaNbV compounds with a body-centered cubic crystal lattice with a pronounced textural orientation along the (200) textural direction were chosen as the objects of study. The choice of research objects was based on the combination of the physicochemical, strength, and thermophysical properties of titanium, tantalum, niobium, and vanadium compounds, which make it possible to create a highly ordered alloy with great potential for use as structural materials for nuclear energy. To obtain the samples, the method of arc melting of chemically pure elements Ti, Ta, Nb, and V was used, and the homogeneity of the alloy was achieved by remelting the composition during five successive procedures. The vacuum in the furnace was at least 5 × 10^−5^ mbar. According to the data of the elemental composition of the obtained alloys, presented in Table 1, it can be concluded that the distribution of elements in the composition of the alloy during its production is equiprobable, which is also evidenced by the mapping results. Data on the elemental composition of the alloy were acquired by evaluating the obtained alloys using the energy-dispersive analysis method implemented on a Hitachi TM3030 scanning electron microscope (Hitachi, Tokyo, Japan). The data were obtained by taking a series of spectra (at least 10 spectra) to determine the average value and measurement error.

### 2.2. Irradiation of Samples and Simulation of Radiation Damage

The samples were irradiated with low-energy He^2+^ ions with an energy of 40 keV in the fluence range 10^15^–5 × 10^17^ ion/cm^2^. Irradiation was carried out at the DC-60 heavy ion accelerator located in the Astana branch of the Institute of Nuclear Physics of the Ministry of Energy of the Republic of Kazakhstan (INP, Astana, Kazakhstan). The choice of the type of low-energy He^2+^ ions for modeling radiation damage was primarily due to the possibility of recreating the processes of formation of gas-filled bubbles in the near-surface layer of ceramics under high-dose irradiation, the formation of which is associated with the low solubility of helium and its high ability to agglomerate when filling voids.

Simulation of radiation damage during irradiation with low-energy He^2+^ ions with an energy of 40 keV was carried out using the SRIM Pro 2013 program code. The Kinchin–Pease model was used for modeling, taking into account cascade interactions. At the same time, the study of the radiation damage mechanisms during irradiation with low-energy He^2+^ ions was carried out in the case of variation of the alloy components. The simulation results were compared with pure niobium.

**Figure 1:**
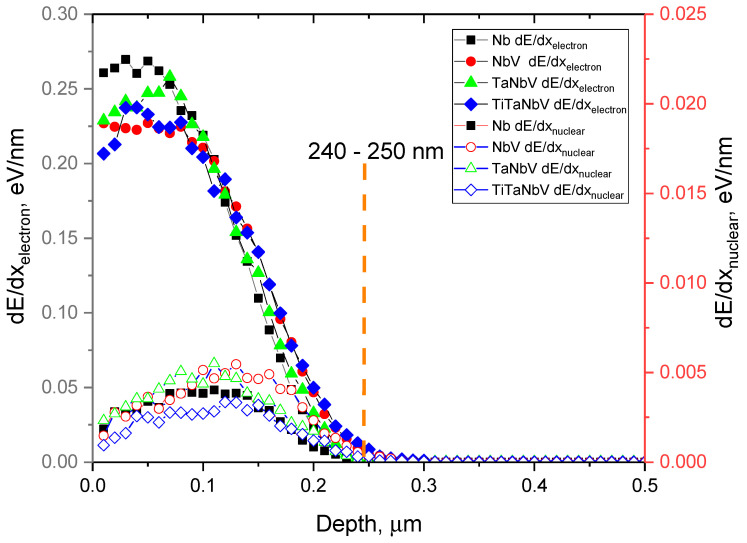
Simulation results of ionization losses of incident He^2+^ ions in TiTaNbV alloys with the variation of the components.

Figure 1 shows the results of estimating the simulation of the ionization losses of incident He^2+^ ions during interaction with the electronic and nuclear subsystems. The general view of the presented dependences of ionization losses characterizes the main processes associated with the interaction of incident ions with the structure, which occurs in the near-surface layer, 200–300 nm thick, in the alloys under study. At the same time, an analysis of the ionization loss values during the interaction of incident ions with electronic and nuclear subsystems showed that the difference between the ionization loss values is more than 5 times, with electronic losses predominating almost along the entire ion trajectory in the material. From which it can be concluded that the greatest contribution to the formation of radiation-induced damage will be made by the results of the interaction of incident ions with the electronic subsystem, causing ionization and as a result, forming metastable states in the electronic subsystem due to ionization processes and subsequent redistribution of the electron density along the ion motion trajectory. It should also be noted that in the range of selected irradiation fluences of 10^15^–5 × 10^17^ ion/cm^2^, the density of accumulated radiation-induced damage will be very significant, which will lead to the possibility of the formation of large agglomerates of defective areas in the near-surface layer, as well as the formation of gas-filled bubbles with an increase in the irradiation fluence. The formation of gas-filled bubbles during high-dose helium irradiation is associated with the high mobility of helium, as well as its low solubility, which makes it possible for it to agglomerate in the voids of the crystal lattice upon implantation into the near-surface layer, followed by accumulation, leading to deformation distortion and swelling [26]. In this case, as shown in several works [27,28], the critical irradiation fluences at which the formation of helium bubbles is observed in materials are 10^17^–5 × 10^17^ ion/cm^2^, which was the motivation for choosing the irradiation range during this experiment.

**Figure 2:**
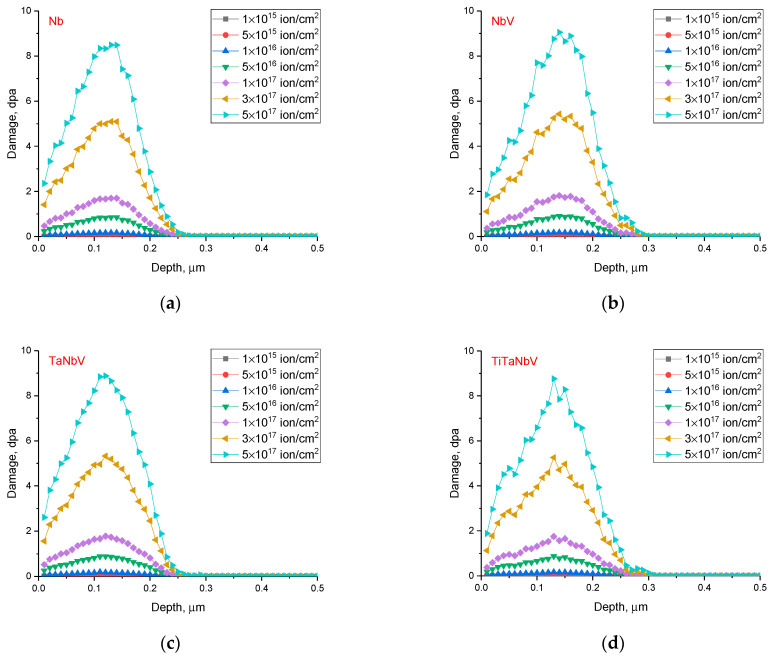
Simulation results of atomic displacements in the alloys under study: (**a**) Nb; (**b**) NbV; (**c**) TaNbV; (**d**) TiTaNbV.

Based on the obtained data on the simulation of ionization losses, the values of atomic displacements in the near-surface layer were calculated depending on the irradiation fluence, which characterizes the degree of deformation of the damaged layer during the radiation damage accumulation. The results of the estimation of atomic displacements (dpa) are presented in Figure 2 for each selected alloy depending on the irradiation fluence.

As can be seen from the presented data of the simulation results of the atomic displacement values, the profile of the change in the values of dpa with respect to the depth of the ion path has a similar character for all the alloys under study when their components are varied. This, in turn, indicates that the accumulation of radiation damage has a single similar character, which has insignificant differences when the alloy components are varied. The maximum dpa value at a fluence of 5 × 10^17^ ion/cm^2^ is no more than 9 dpa, while for fluences below 10^17^ ion/cm^2^ the value of atomic displacements is less than 1 dpa.

### 2.3. Measurement of Structural Distortions and Morphological Features of the Surface of Alloys as a Result of Irradiation

The study of structural features and their changes as a result of the radiation damage accumulation in the near-surface layer was carried out by analyzing the obtained X-ray diffraction patterns of the studied samples before and after irradiation, as well as a comparative analysis of changes in the parameters and volume of the crystal lattice. X-ray diffraction patterns were taken using a D8 Advance ECO powder X-ray diffractometer (Bruker, Berlin, Germany). The diffraction patterns were obtained in the Bragg–Brentano geometry in the angular range 2θ = 30–100⁰. X-ray studies were carried out considering the comparison of irradiated samples with non-irradiated samples to determine the structural changes caused by irradiation. Additionally, when taking diffraction patterns, a system of sollers was used, which made it possible to reduce the beam intensity, and the power of the tube was selected in such a way that the main data were obtained from the damaged layer.

### 2.4. Evaluation of Strength Properties and Thermal Stability of Irradiated Specimens

The strength properties of the alloys and their change depending on the irradiation fluence were evaluated by indentation using a LECO 700 microhardness tester (Leco Corporation, St. Joseph, Michigan, USA). The indentation conditions were chosen in such a way that the indenter penetration depth did not exceed the ion path depth in the material. To do this, small loads on the indenter, the value of which was 0.5 N, were used. A Vickers pyramid was used as an indenter. To determine the hardness of the near-surface layer depending on the irradiation fluence and standard deviation values, all measurements were performed serially (25 measurements at different points on the surface). The calculation of the softening degree depending on the radiation damage degree was performed on the basis of a comparative analysis of the hardness values before and after irradiation.

The evaluation of the thermal stability of irradiated samples, as well as the effect of accumulated radiation damage on the stability of strength properties during cyclic tests, was carried out as follows. The samples, after irradiation, were heated to a temperature of 1000 °C at a heating rate of 20 °C/min, held for 1 h, and then cooled for 10 h. This procedure continued for 20 successive cycles, after each of which the strength characteristics of the alloys were measured and the softening values were determined. The choice of these temperatures for heat resistance testing was due to the possibility of using these alloys in operating conditions at elevated temperatures (more than 500 °C), as well as possible crisis situations arising from the overloading of installations and a sharp increase in operating temperature.

## 3. Results and Discussion

### 3.1. Characterization of the Structural and Strength Properties of Alloys Depending on the Variation of the Alloy Components

**Figure 3:**
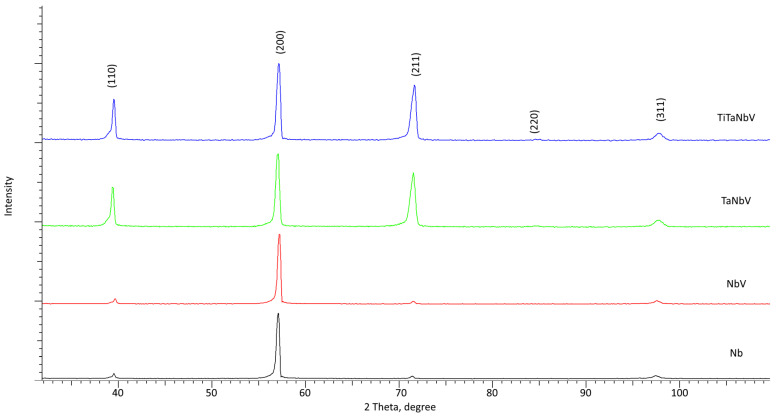
Results of X-ray phase analysis of the studied alloys.

Figure 3 shows the results of the X-ray phase analysis of the alloys under study with variations of the alloy components, reflecting not only the change in the phase composition but also the structural parameters. The overall view of the presented diffraction patterns is typical for structures with a body-centered cubic lattice, which is characteristic of alloys based on Nb–V–Ta–Ti compounds. At the same time, variation of alloy components leads to a change in both the shape and position of diffraction reflections and their intensity. Variation in the shape of reflections and shift in the diffraction maxima position with a change in the alloy components is due to the substitution of atoms in the lattice sites and the difference in the values in the atomic radii of niobium (146 pm), vanadium (134 pm), tantalum (149 pm), and titanium (147 pm) [29]. A change in the composition of alloys leads to the appearance of tensile or compressive distortions of the crystal lattice due to the substitution of atoms at the crystal lattice nodes. In this case, the shift of the maxima of diffraction reflections unambiguously depends on the variation of the components and atomic radii, which also affects the changes in the crystal lattice parameters and deformation factors associated with their deviations in comparison with the reference values. The presence of slight changes in the shape of the (110) and (211) diffraction maxima associated with the asymmetry of the shape of the reflections can be characteristic of substitution effects when varying the alloy components, as well as the formation of small inclusions of unidentifiable phases in the form of single cubic compounds. At the same time, these changes are most pronounced in TaNbV and TiTaNbV alloys. In this case, the addition of tantalum and, subsequently, titanium to the NbV alloy leads to an increase in the intensities of the (110) and (211) diffraction reflections, which indicates the effects of grain textural reorientation during the formation of alloys in the case of an increase in the number of components. In this case, the distortion of the shape of reflections (110) and (211) indicates a deformation distortion of the crystal lattice, with a variation of the alloy components.

Table 2 shows the results of the evaluation of the structural parameters for the studied alloys depending on the variation of the components, reflecting changes in the crystal structure.

As can be seen from the data presented in Table 2, a change in the alloy components leads to a decrease or increase in the parameters and volume of the crystal lattice, which is due to the difference in the ionic radii of the selected components. At the same time, the analysis of the deformation factor of distortions of the crystal lattice showed that in the case of the synthesis of an alloy from niobium, the technology used leads to the greatest deformation of the crystal lattice in comparison with other types of alloys. Additionally, in the case of adding vanadium to the alloy, there is a change in the type of deformation distortions from tensile to compressive (sign change in the obtained value). This can be due both to the effect of the difference in ionic radii, which leads to a decrease in the parameters of the crystal lattice and, accordingly, to its compression and to denser packing of the crystal lattice. It should also be noted that the addition of various components to the alloy leads to a decrease in the deformation factor by an order of magnitude, which indicates that the formation of high-entropy alloys occurs with denser packing of the crystal lattice, as well as a decrease in structurally distorted regions in the material.

**Figure 4:**
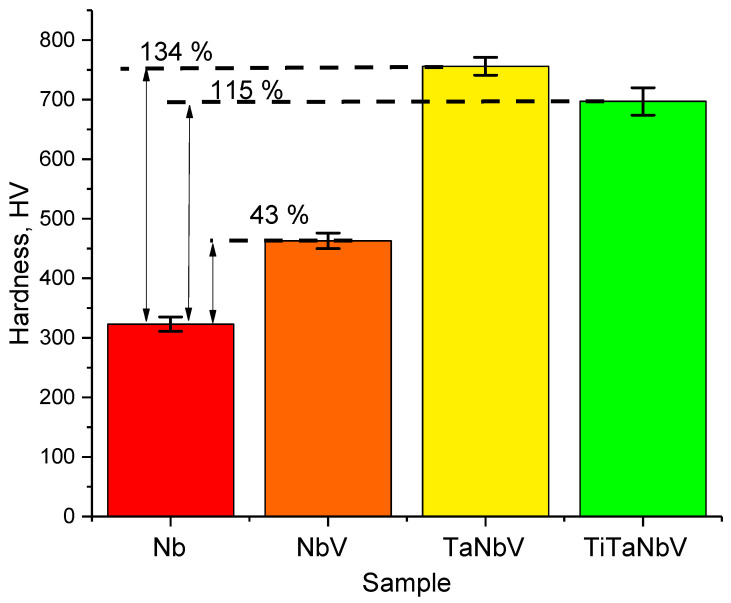
Diagram comparing the change in the hardness value of HEAs depending on the variation of the alloy components.

Figure 4 shows the results of changes in hardness values depending on the variation of the components of an alloy, reflecting the hardening factor with a change in composition.

As can be seen from the data presented, the variation of the alloy components by adding vanadium and tantalum to it leads to an increase in hardness by 43 and 135% compared to similar values for pure niobium. At the same time, the addition of titanium to the alloy leads to a slight (less than 20%) decrease in hardness compared to the value for the TaNbV alloy. This reduction is due to the effects of titanium’s lower density resulting in a reduction in hardness; however, the addition of titanium makes it possible to lighten the alloy and increase its resistance to corrosion and degradation. The effects of hardening upon variation of the alloy composition are primarily due to the strength properties of refractory metals, as well as the formation of a mixed equimolar composition, the variation of the components, which leads to the formation of a high-strength composition.

### 3.2. Effect of Irradiation on Structural Distortions of the Near-Surface Layer of Alloys during Irradiation

**Figure 5:**
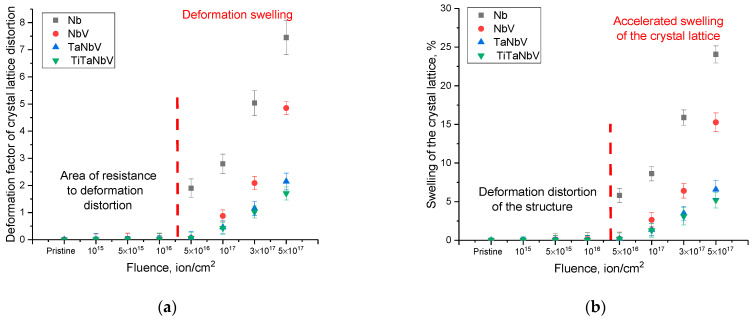
(**a**) Change in the deformation factor of distortion of the crystal lattice of alloys depending on the irradiation fluence. (**b**) Swelling of the crystal lattice depending on the irradiation fluence.

Figure 5 shows the results of the evaluation of structural distortion (deformation factor) and crystal lattice swelling as a result of irradiation with He^2+^ ions with different fluences. An analysis of the obtained data on the deformation factor of crystal lattice distortions showed that the main deformations are observed at high irradiation fluences (above 10^16^ ion/cm^2^), which indicates a fairly high resistance of the alloys under study to near-surface radiation-induced destruction. In this case, changes in structural parameters below a fluence of 10^16^ ion/cm^2^ can be due to deformation distortions of the crystal lattice during the interaction of incident ions with the material; however, due to the high stability of alloys, most of the resulting structural distortions annihilate. At the same time, the nature of deformation distortions indicates that the main type of structural distortions is associated with tensile deformations caused by the accumulation of radiation damage (an increase in the magnitude of atomic displacements), as well as agglomeration of implanted helium during high-dose irradiation. According to the calculated data on the concentration of atomic displacements in the near-surface layer of alloys, it was found that at irradiation fluences above 10^16^ ion/cm^2^, the displacements are at least 2–9 dpa, depending on the irradiation fluence. At such displacements, the dominant role in the structural distortions of the crystal lattice is played by deformations caused by primary knocked-on atoms that can migrate over the damaged layer. At the same time, at high irradiation fluences (above 10^17^ ion/cm^2^), structural distortions also occur due to implanted helium in the near-surface layer, which, due to its high mobility and ability to agglomerate, can fill voids formed as a result of knocked-out atoms, thereby forming gas-filled regions. A rise in the implanted helium concentration leads to an increase in the size of these gas-filled regions, which, growing in size, exerts tensile stress on the crystal lattice, leading to its destabilization.

The presence in the composition of alloys of various elements that form a solid solution leads to a decrease in the rate of accumulation of structural distortions, which is expressed in lower values of the deformation factor of distortions, as well as the magnitude of swelling. In this case, the accumulation of structural distortions means the agglomeration of radiation-induced defects, as well as implanted He^2+^ ions, the introduction of which can lead to a destructive change in the crystal lattice. Moreover, the presence of structural distortions caused by variations in the composition of high-entropy alloys can play a dual role in changing the properties of irradiated materials. On the one hand, structural distortions caused by variations in alloy composition can serve as additional obstacles for migrating defects and implanted He^2+^. On the other hand, these structural distortions can be accumulating centers where point defects and vacancies will accumulate, and in the case of implanted He^2+^ accumulation, gas-filled bubbles can form.

Similar effects of deformation distortion of the crystal lattice have been established in several studies aimed at studying radiation damage kinetics in high-entropy alloys and steels [30,31,32]. At the same time, in a review [30] devoted to the study of radiation damage in high-entropy alloys, an explanation is given for the effects of lower structural distortions of the crystal lattice for multicomponent alloys associated with the difference in the ionic radii of various components of the alloy, as well as the irregular arrangement of atoms at the lattice sites. Such an irregular arrangement of atoms in the crystal lattice can lead to additional effects associated with slowing down the motion of primary knocked-on atoms, implanted ions, and dislocations. As a result, the rate of structural deformation decreases due to the inhibition of the diffusion migration of defects and implanted ions.

As can be seen from the data presented, an increase in the alloy components leads to a decrease in the effect of swelling of the crystal lattice of the near-surface layer, as well as its distortion at high irradiation fluences. Such a decrease in radiation damage during the accumulation of implanted helium can be due to the following factors. In a study on the determination of the stability to helium swelling of high-entropy alloys, the authors of [17] showed that high-entropy alloys are more resistant to destruction than austenitic alloys. The authors attribute the increase in stability to slowing down the processes of agglomeration and subsequent swelling of the near-surface damaged layer due to structural distortions, leading to the creation of additional barriers. At the same time, the authors of [33] showed that the degradation of the damaged layer occurs as a result of the formation of fragmentary dislocation loops, which, when the composition of the high-entropy alloy is varied, cannot form complex defects. This can be explained by the effects associated with the high entropy of mixing the composition of the alloys, which creates additional obstacles to destruction in the structure.

**Figure 6:**
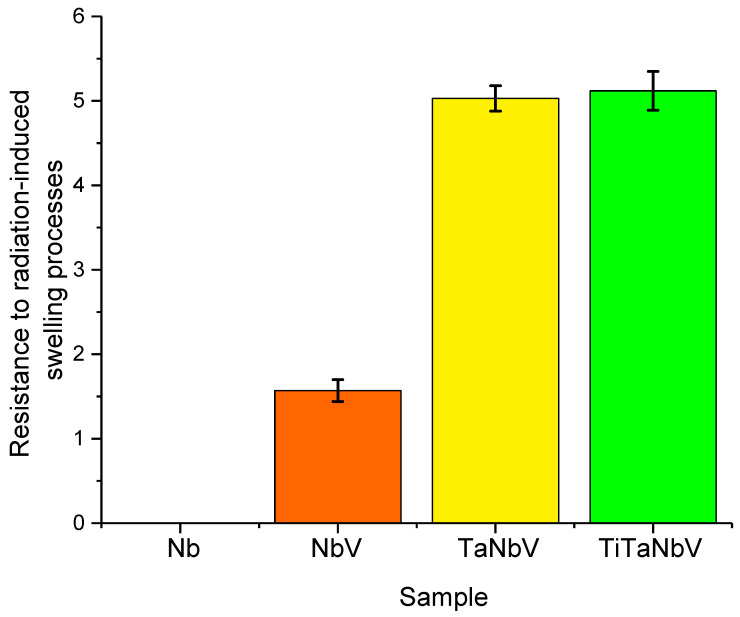
Comparison of the value of resistance to radiation-induced swelling due to irradiation.

An analysis of the data presented in Figure 5b, reflecting changes in the volume of the crystal lattice, indicates an increase in the resistance of alloys to volume swelling as a result of the accumulation of deformation distortions in the case of an increase in the number of alloy components. In the case of the Nb alloy, the maximum swelling is more than 25%, which indicates a strong destruction of the crystal structure and its partial amorphization. At the same time, for TaNbV and TiTaNbV alloys, the maximum swelling of the crystal lattice is no more than 5–6%, which is five times less than the similar swelling value for the Nb alloy. Based on the obtained data on the change in deformation distortions and volumetric swelling of the crystal lattice, it can be concluded that the resistance to radiation-induced swelling caused by irradiation has increased (see Figure 6 data).

As can be seen from the data presented, in comparison with the Nb alloy, with the addition of vanadium, the resistance increases by 1.5 times, while the addition of tantalum and titanium leads to a five-fold increase in the resistance to swelling. Such behavior of the structure of alloys to the processes of radiation-induced swelling can be associated both with the effects of an increase in the density of alloys with the variation of the components, as well as the presence of a large number of interstices and vacancies at the grain boundaries, which leads to the appearance of the recombination effect, as well as an increase in the resistance to swelling [34].

### 3.3. Change in Strength Properties as a Result of Radiation Damage Accumulation in the Near-Surface Layer

**Figure 7:**
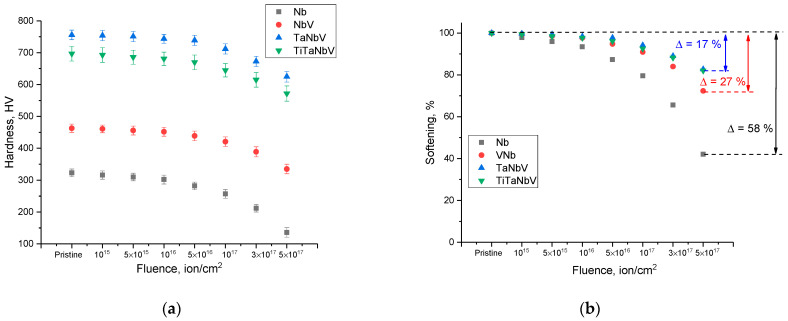
(**a**) Results of changes in the hardness of the near-surface layer of the studied alloys depending on the irradiation fluence. (**b**) Results of the softening of alloys during the radiation damage accumulation.

Figure 7a shows the data on changes in the hardness of the near-surface layer of alloys depending on the irradiation fluence, reflecting the softening of the near-surface layer during the accumulation of radiation damage and the consequences associated with them. The overall view of the presented dependences of the change in the values of the hardness of the near-surface layer indicates the differing nature of the strength changes in the alloys associated with the effects of hardening with a variation in the composition of alloys, as well as the degree of structural distortions caused by irradiation. In this case, the dependence of the change in hardness values has a pronounced dependence on the irradiation fluence and, as a consequence, on the value of the accumulated radiation damage. An analysis of the obtained data showed that the main changes in the hardness in the case of pure niobium are observed at an irradiation fluence above 10^16^ ion/cm^2^. While for two–four component alloys, softening occurs at fluences above 5 × 10^16^ ion/cm^2^. This indicates that the rate of radiation damage accumulation and related softening effects decreases with an increase in the alloy components.

The dependences of the softening of the near-surface layer of alloys as a function of the irradiation fluence presented in Figure 7b indicate the effect of the accumulation of radiation damage on the degradation of strength properties. The softening value was calculated on the basis of data on changes in the hardness of alloys depending on the irradiation fluence in comparison with the initial value of hardness for the studied samples subjected to irradiation.

According to the data presented, the greatest changes associated with softening are observed for niobium alloy samples, the decrease in hardness which was more than 55% compared to the initial value. In this case, the variation of the alloy components leads to a more than a 2–3-fold increase in the resistance to softening caused by the radiation damage accumulation. In the case of TaNbV and TiTaNbV alloys, the softening value is no more than 17% for both alloys, which indicates the same resistance to degradation, despite the fact that in the initial state, the hardness of the TaNbV alloy is higher. Such a difference in the strength properties of alloys, in particular, resistance to hardness degradation during the accumulation of radiation damage, can be explained by differences in structural distortions and deformations of the crystal lattice caused by irradiation, as well as a difference in the accumulation of helium inclusions in the structure of the near-surface layer, leading to the formation of gas-filled bubbles.

**Figure 8:**
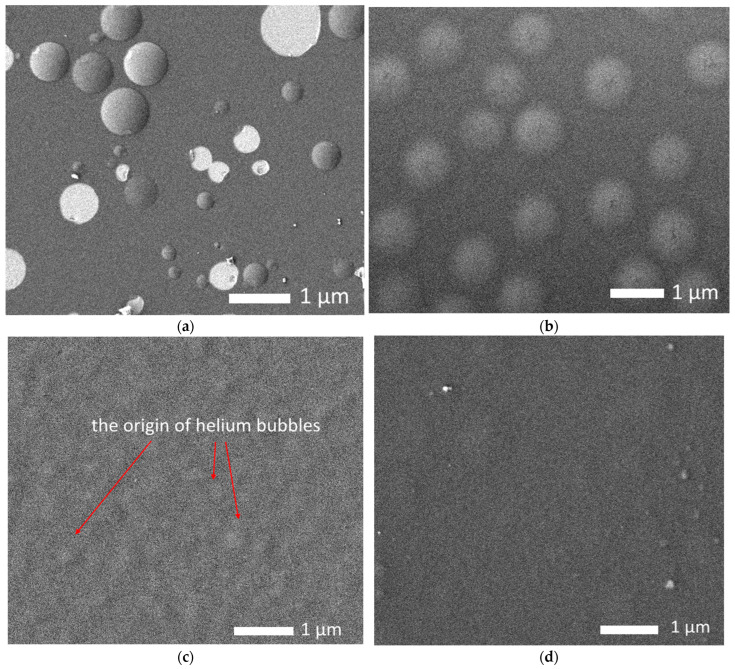
Results of morphological changes in the surface of alloys after irradiation with He^2+^ ions with a fluence of 5 × 10^17^ ion/cm^2^: (**a**) Nb; (**b**) NbV; (**c**) TaNbV; (**d**) TiTaNbV.

Figure 8 shows the results of morphological studies of the effect of helium swelling on the near-surface layer of alloys after irradiation. These images were obtained for samples irradiated with a fluence of 5 × 10^17^ ion/cm^2^.

As can be seen from the presented SEM images, in the case of the Nb alloy, the formation of large helium inclusions was observed on the surface, some of which were destroyed. This indicates that the accumulation of structural distortions leads to the destruction of gas-filled bubbles, resulting in explosive destruction of the near-surface layer and partial exfoliation of the surface. This nature of the destruction of gas-filled bubbles is due to the fact that at an increased concentration of helium in the filled area, the deformation distortion leading to the tension of the gas-filled cavity increases, as a result of which destruction occurs, followed by the explosive opening of the bubble and destruction of the area. At the same time, part of the near-surface layer is embrittled, which leads to a decrease in strength properties. The presence of gas-filled bubbles of various diameters, including destroyed areas, indicates that the swelling mechanisms have different rates associated with the migration and agglomeration of implanted helium in the near-surface layer, which is due to the presence of structural defects and their distortion.

When vanadium is added to the composition of the alloy, the presence of bubbles is observed on the surface of the studied samples, which also indicates that helium accumulation is observed in the structure of the near-surface layer, followed by the formation of gas-filled inclusions. However, unlike the Nb alloy, no open bubbles were observed on the surface of the NbV alloy, which indicates an increased resistance of the near-surface layer to helium swelling. In the case of the TaNbV alloy, swellings were observed on the surface, which are the nuclei of helium bubbles; however, their density and diameters are much lower than for the NbV alloy, which indicates an increased resistance to helium swelling, which is due to the high resistance of the crystal structure to deformation distortions. In the case of the TiTaNbV alloy, no inclusions typical of gas-filled bubbles were observed on the surface, which indicates the resistance of these alloys to destructive embrittlement. From the analysis of the observed morphological changes depending on the variation of the alloy compositions, it can be concluded that an increase in the alloy components leads to increased resistance to helium swelling and subsequent destructive embrittlement of the near-surface layer.

### 3.4. Study of Thermal Stability of Samples before and after Irradiation

**Figure 9:**
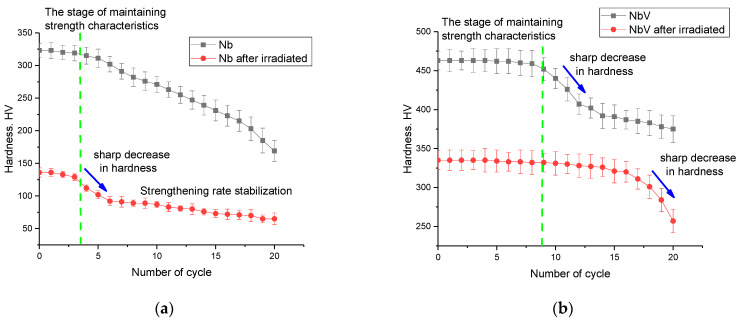

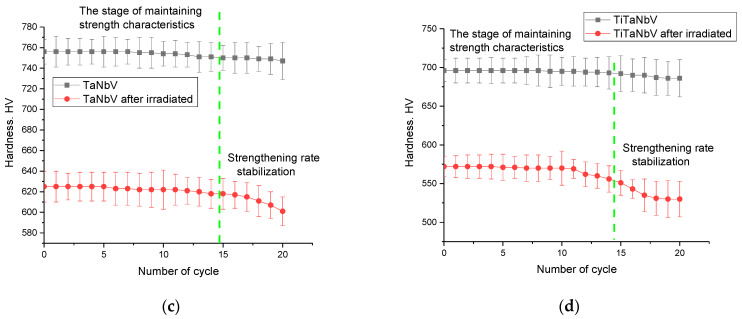
Results of the assessment of changes in the hardness values of the studied samples as a result of tests for thermal stability: (**a**) Nb; (**b**) NbV; (**c**) TaNbV; (**d**) TiTaNbV.

Figure 9 shows the results of estimating the hardness parameters of the alloys under study depending on the number of thermal stability test cycles during heating and subsequent cooling. The data are presented as a comparison of changes in the hardness values of the samples in the initial and irradiated (5 × 10^17^ ion/cm^2^) states in order to determine the effect of radiation damage on the thermal stability of the samples.

As can be seen from the presented data of cyclic dependences of the change in the hardness of the samples, the general form of the changes is characterized by two characteristic areas: the stable preservation of the strength characteristics during testing and the decrease in hardness values, indicating a destructive change in strength. At the same time, the stage of stable preservation of strength is quite different for samples of different compositions. In the case of the Nb alloy, the stability of the strength characteristics, both in the case of the original sample and the irradiated one, is no more than 3–4 successive cycles, after which a decrease in strength is observed. At the same time, it should be noted that the nature of the decrease in strength characteristics for the irradiated sample has two characteristic areas with different rates of strength degradation, which indicates that a destructive change in the near-surface layer, accompanied by a sharp deterioration in properties and subsequent stabilization of the decrease in strength. Such a sharp change may be due to the effects associated with an increase in the mobility of small vacancies and helium bubbles in the structure of the damaged layer, which leads to a sharp increase in the size of large gas-filled regions due to the agglomeration of implanted helium. An increase in the size of gas-filled bubbles can lead to uncontrolled swelling and subsequent destruction, which will lead to embrittlement and exfoliation of the surface of alloys. Additionally, in the case of the Nb alloy, a sharp deterioration in the strength characteristics can be associated with oxidation processes during heat treatment with the formation of a porous oxide film on the surface of the samples.

In the case of NbV, there was a difference in the dynamics of hardness change during cyclic tests for irradiated and non-irradiated samples. For non-irradiated samples, stable retention of strength characteristics was observed for 7–8 successive cycles, with a sharp deterioration in hardness and subsequent stabilization of degradation after 13 successive cycles. This behavior of the strength characteristics for the original sample may be due to an increase in the resistance to oxidation due to the partial replacement of niobium with vanadium, which leads to stabilization and an increase in resistance to high-temperature corrosion. At the same time, for irradiated samples, the presence of deformation distortions caused by irradiation leads to an increase in the area of stability of strength characteristics up to 15 successive cycles, followed by a sharp change in strength characteristics.

For TaNbV and TiTaNbV alloys, the change in strength characteristics as a result of thermal tests in the case of initial samples was practically not observed, and the decrease in hardness values was within the measurement error. At the same time, for irradiated samples, a change in hardness during thermal tests was observed after 15 cycles, which indicates a sufficiently high resistance to the destruction of irradiated alloys during high-temperature stress tests. In turn, the decrease in hardness after 15 cycles of successive tests can be due to the migration processes of implanted helium in the near-surface layer, as well as vacancies and interstitial atoms, which in turn leads to agglomeration and subsequent swelling due to the formation of gas-filled inclusions.

**Figure 10:**
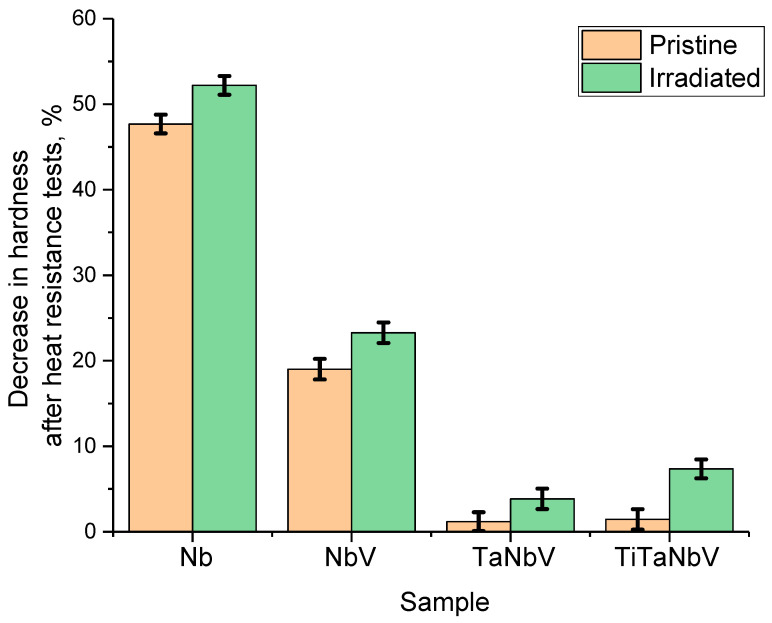
Results of a comparative analysis of changes in the hardness values of samples after tests for thermal stability of samples in the initial and irradiated state.

Figure 10 shows the results of a comparative analysis of changes in the hardness values of samples after testing for the thermal stability of samples after 20 successive cycles. The presented data testify to the effect of alloy softening after life tests as a result of thermal heating and subsequent cooling, simulating stress temperature loads. At the same time, the analysis of these changes in hardness indicates that the high-temperature destruction of the strength characteristics occurs both in the original samples and in the irradiated samples but at a lower rate. In the case of Nb alloy, in the case of a non-irradiated sample, the decrease in hardness is more than 45%, while for an irradiated sample, this value is more than 50%, which indicates greater destruction of the damaged layer. At the same time, unlike other Nb alloys, the alloy showed the worst resistance to thermal stability tests, the deterioration of properties of which was 2.5–7 times higher than the results for NbV, TaNbV, and TiTaNbV alloys. In the case of an NbV alloy, the deterioration in strength properties is no more than 17–20%, and the difference between the original and irradiated specimen is no more than 2–3%. This indicates that deformation distortions and, as a result, the accumulation of radiation damage in the composition of alloys affect the resistance to thermal tests. In the case of irradiated TaNbV and TiTaNbV alloys, the destructive change in strength properties is no more than 5 and 10%, respectively. At the same time, the change in the strength properties of non-irradiated samples of alloys as a result of 20 consecutive tests for thermal stability was no more than 2%, which indicates a high resistance of these alloys to thermal stress tests.

**Figure 11:**
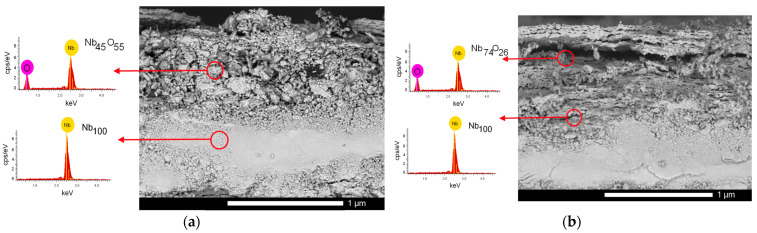
Results of evaluation of lateral cleavages of Nb alloy after life tests for thermal stability in the initial (**a**) and irradiated states (**b**).

One of the explanations for such a sharp deterioration in the hardness of the Nb alloy as a result of thermal stability tests may be the oxidation of the near-surface layer with the formation of a porous oxide film, which leads to accelerated destruction of the alloy surface and a decrease in its strength properties. Figure 11 shows the results of the evaluation of the side profile of the Nb alloy after thermal stability tests, which reflect the formation of an oxide film as a result of the thermal heating of the samples. As is known, metallic niobium has a low oxidation temperature in an oxygen-containing environment (less than 600 °C) with the formation of an oxide film on the surface, the thickness of which increases with increasing annealing time or temperature. As can be seen from the data presented, in the case of Nb alloy samples after thermal testing, the surface is a mixture of an oxide film, the thickness of which is at least 500–700 nm in the case of the original sample and somewhat less (no more than 500 nm) in the case of the irradiated sample. At the same time, the structure of this oxide film for the original sample and the irradiated one has obvious differences. In the case of the initial sample, the oxidized layer is represented by a mixture of grain inclusions that make up the porous film, while for the irradiated samples, the oxide film delamination is observed with the formation of extended voids. When vanadium, tantalum, and titanium were added to the niobium alloy, the formation of oxide inclusions was not observed as a result of high-temperature tests. This absence of oxide inclusions is due to the effect of replacing niobium with vanadium, tantalum, and titanium and increasing the oxidation resistance of the alloys. The presence of such voids may be due to the effects of helium swelling and subsequent accelerated destruction observed in the case of increased migration under temperature exposure. As is known, heat treatment of steels, ceramics, or alloys subjected to helium irradiation leads to the initialization of helium agglomeration processes, followed by an increase in the size of gas-filled cavities, as well as their enlargement [35,36,37]. As a result, thermal testing of irradiated samples, in addition to oxidation processes accompanied by structural changes in the damaged layer, can lead to destructive embrittlement of the surface when gas-filled cavities are opened, followed by their destruction and exfoliation.

**Figure 12:**
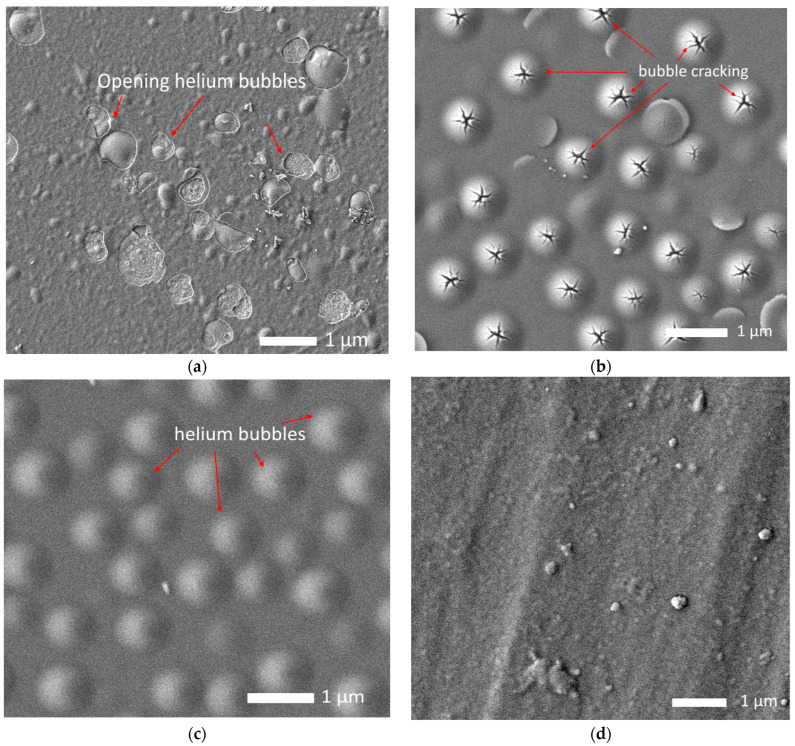
Results of morphological changes in the surface of alloys after irradiation with He^2+^ ions with a fluence of 5 × 10^17^ ion/cm^2^ and after cyclic tests for thermal stability: (**a**) Nb; (**b**) NbV; (**c**) TaNbV; (**d**) TiTaNbV.

Figure 12 shows the results of morphological studies of alloys after thermal stability tests after 20 successive test cycles.

As can be seen from the presented data, in the case of irradiated Nb alloy samples after cyclic high-temperature tests, an increase in the destructive destruction of the near-surface layer is observed due to explosive exfoliation of damaged gas-filled regions, as well as the formation of small helium bubbles, indicating accelerated migration processes of implanted helium to the surface, followed by the formation of new bubbles near the destroyed areas. In the case of the NbV alloy, the decrease in strength characteristics can be due to the effects of the partial opening of gas-filled bubbles associated with an increase in deformation distortions caused by an increase in pressure in the filled regions. Additionally, the accelerated migration of implanted helium in the composition of the damaged layer of alloys is evidenced by the appearance of gas-filled bubbles in the surface layer of the TaNbV alloy, as well as deformation distortions of the surface of the TiTaNbV alloy. However, in the case of TaNbV and TiTaNbV alloys, no partial opening of gas-filled inclusions or their exfoliation was observed, which indicates that the migration processes of implanted helium are hindered in these alloys due to the presence of structural distortions of the crystal structure associated with the multicomponent nature of the alloys.

## 4. Conclusions

During experimental work, the mechanisms of accumulation of radiation damage in alloys based on Nb-V-Ta-Ti compounds with varying alloy components, as well as their effect on changes in deformation distortions and strength properties of alloys, were established. It was established that a change in the concentration of the alloy components due to the addition of tantalum and titanium leads to a decrease in the deformation distortions of the crystal structure and its bulk swelling in comparison with the Nb alloy. During the evaluation of the strength characteristics, it was found that an increase in the irradiation fluence above 10^16^ ion/cm^2^ leads to a decrease in the strength properties, the change of which is associated with the accumulation of deformation distortions leading to the formation of deformation-saturated inclusions, as well as cavities filled with implanted helium. In the case of tests for high-temperature corrosion of initial and irradiated samples during thermal annealing and subsequent cooling during cyclic tests, it was found that the Nb alloy has the lowest stability to high-temperature heating, the degradation of which is associated with the formation of a porous oxide layer on the surface of the sample. It was also found that the formation of radiation-induced distortions upon variation of the alloy components leads to a decrease in the diffusion rate of implanted helium and difficulties in agglomeration, and the formation of gas-filled inclusions.

## Figures and Tables

**Table 1:** Elemental composition data.

Sample	Concentration of Element, at. %			
	Nb	V	Ta	Ti
Nb	100	-	-	-
NbV	48.3 ± 1.5	51.7 ± 1.4	-	-
TaNbV	32.2 ± 1.2	34.1 ± 1.3	33.7 ± 1.6	-
TiTaNbV	24.6 ± 1.1	27.3 ± 0.8	24.7 ± 1.2	23.4 ± 1.5

**Table 2:** Data of structural parameters of the studied alloys.

Parameter	Alloy			
	Nb	NbV	TaNbV	TiTaNbV
Lattice parameter, Å	a = 3.2231 ± 0.0025	a = 3.2182 ± 0.0016	a = 3.2261 ± 0.0018	a = 3.2205 ± 0.0026
The volume of the crystal lattice, Å^3^	33.48	33.33	33.58	33.40
Deformation factor of crystal lattice distortion	0.024	−0.012	0.0012	0.0029

## Data Availability

Not applicable.
